# The modulation of macrophage subsets in celiac disease pathogenesis

**DOI:** 10.1002/iid3.741

**Published:** 2022-11-16

**Authors:** Sara Molaaghaee‐Rouzbahani, Nastaran Asri, Somayeh Jahani‐Sherafat, Davar Amani, Andrea Masotti, Kaveh Baghaei, Abbas Yadegar, Hamed Mirjalali, Mohammad Rostami‐Nejad

**Affiliations:** ^1^ Department of Immunology, School of Medicine Shahid Beheshti University of Medical Sciences Tehran Iran; ^2^ Gastroenterology and Liver Diseases Research Center, Research Institute for Gastroenterology and Liver Diseases Shahid Beheshti University of Medical Sciences Tehran Iran; ^3^ Laser Application in Medical Sciences Research Center Shahid Beheshti University of Medical Sciences Tehran Iran; ^4^ Bambino Gesù Children's Hospital‐IRCCS Research Laboratories Rome Italy; ^5^ Foodborne and Waterborne Diseases Research Center, Research Institute for Gastroenterology and Liver Diseases Shahid Beheshti University of Medical Sciences Tehran Iran

**Keywords:** autoimmune disease, celiac disease, gluten, inflammation, intestinal disease, macrophages, tissue homeostasis

## Abstract

**Background:**

So far, limited studies have focused on the role of Macrophages (MQs) in the development or progression of celiac disease (CD). Researchers believe that increasing knowledge about the function of MQs in inflammatory disorders plays a critical role in finding a new treatment for these kinds of diseases.

**Main body:**

CD is a permanent autoimmune intestinal disorder triggered by gluten exposure in predisposed individuals. This disorder happens due to the loss of intestinal epithelial barrier integrity characterized by dysregulated innate and adaptive immune responses. MQs are known as key players of the innate immune system that link innate and adaptive immunity. MQs of human intestinal lamina propria participate in maintaining tissue homeostasis, and also intestinal inflammation development. Previous studies suggested that gliadin triggers a proinflammatory phenotype (M1 MQ) in human primary MQs. Moreover, M2‐related immunosuppressive mediators are also present in CD. In fact, CD patients present an impaired transition from pro‐inflammatory to anti‐inflammatory responses due to inappropriate responses to gliadin peptides.

**Conclusion:**

The M1/M2 MQs polarization balancing regulators can be considered novel therapeutic targets for celiac disease.

## Graphical abstract

Celiac disease (CD) is a permanent autoimmune intestinal disorder triggered by gluten exposure in predisposed individuals. Macrophages of human intestinal lamina propria participate in maintaining tissue homeostasis, and also intestinal inflammation development. Due to the importance of macrophages in CD pathogenesis, as well as the importance of finding a new therapeutic approach for this disorder, we discussed the current knowledge about the role of macrophage subset modulation in CD pathogenesis.

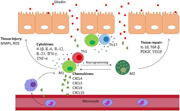

## 1 INTRODUCTION

Celiac disease (CD) is a chronic autoimmune disorder of the small bowel that affects approximately 1% of the population around the world.^1^, ^2^, ^3^ This disorder is caused by abnormal immune responses to gluten proteins (a mixture of gliadins and glutenins), which are found in certain cereal grains like wheat, rye, and barley.^1^, ^4^, ^5^ Gluten is enriched in glutamine and proline amino acids and is incompletely digested in the gastrointestinal (GI) tract leading to a remaining fraction of 33‐mer peptides, which is highly toxic for CD patients.^6^ In fact, gluten, environmental factors, and host genetic makeup are involved in CD pathogenesis. The highest risk of CD development is found in subjects with HLA DQ2 and HLA DQ8 haplotypes.^7^, ^8^ Vitamin D rate, breast‐feeding, intestinal infection, and gut microbiota alterations are among environmental risk factors that can cause CD development.^6^, ^9^ In fact, CD happens as a result of alteration in intestinal epithelial barrier function and innate and adaptive immune responses activation, leading to villous atrophy and crypt hyperplasia.^10^

Macrophages (MQs) are mononuclear phagocytes known as key players of the innate immune system that link innate and adaptive immunity. These cells are the first immune cells to emerge in the developing embryo and act as a defensive agent against pathogens and other environmental challenges and have a role in tissue and immune homeostatic maintenance.^11^, ^12^ The majority of MQs are located throughout the lamina propria (LP) of the GI tract mucosa due to the need to maintain tolerance to an unlimited array of antigens like dietary proteins, toxins, commensal microbiota, and so forth, and protect intestinal homeostasis.^12^ In line with this housekeeping function, they produce soluble factors like prostaglandin E2 (PGE2) that are useful in supporting epithelial barrier integrity.^13^ They can also prevent excessive inflammation in response to harmless antigens by inducing the expansion of CD4+ CD25+ regulatory T (Treg) cells.^14^ However, during intestinal inflammation MQs become highly responsive and induce pro‐inflammatory cytokines secretion and regulate the inflammatory responses.^15^ Previous studies suggested that these cells drive antigen‐specific immune responses to gliadin peptides in CD patients.^10^ Palman et al.^16^ in their case report study observed MQ activation syndrome (a clinical syndrome of hyperinflammation) which was triggered by CD. Researchers believe that studying MQs' function in inflammatory disorders aid our understanding and finding of a new treatment for these kinds of diseases.

Until now, lifelong complete adherence to a gluten‐free diet (GFD) is the only proven treatment for CD patients. However, in practice, this diet is difficult to follow for a long time and has a financial burden for patients.^17^ Therefore, finding an alternative novel therapeutic strategy for CD patients becomes the subject of many studies.^18^, ^19^ One of the therapeutic approaches, which is under study, is using Tolerogenic immune‐modifying nanoparticles (TIMP) that can induce immune tolerance to gliadin peptides through interaction with MARCO (macrophage receptor with collagenous structure) expressing MQs.^20^ The phase 1 and 2 clinical trials for this nanoparticle are ongoing with hopeful results.^20^ It points to the importance of MQs in CD, something that little research has addressed so far. In this review paper, we aimed to discuss the current knowledge of MQs development and their role in CD pathogenesis.

## 2 MACROPHAGE CELLS

MQs, white blood cells which were first discovered in the 19th century by Ilya Metchnikoff, are known as the main players of the innate immune system that are necessary for mediating intestinal homeostasis, host defense, and injury response.^21^ During organogenesis, MQs originate from the embryonic yolk sac and fetal liver precursors and after birth and following the occurrence of inflammatory reactions, bone marrow monocytes (Mo) infiltrate into inflamed tissues and differentiate into MQs or monocyte‐derived DCs. According to the last reports, embryonic‐derived MQs have an important role in tissue homeostasis regulation and bone marrow‐derived MQs are important in host defense reactions and inflammatory diseases.^11^ According to single cell RNA sequencing (scRNA‐seq) based studies, MQs are distributed in almost all tissues of the body (like liver, brain, bones, etc.) and play a distinct function in each of them.^22^ Tissue MQs are non‐migratory cells that monitor their close surroundings and obtain different effector functions.^23^

**Figure 1:**
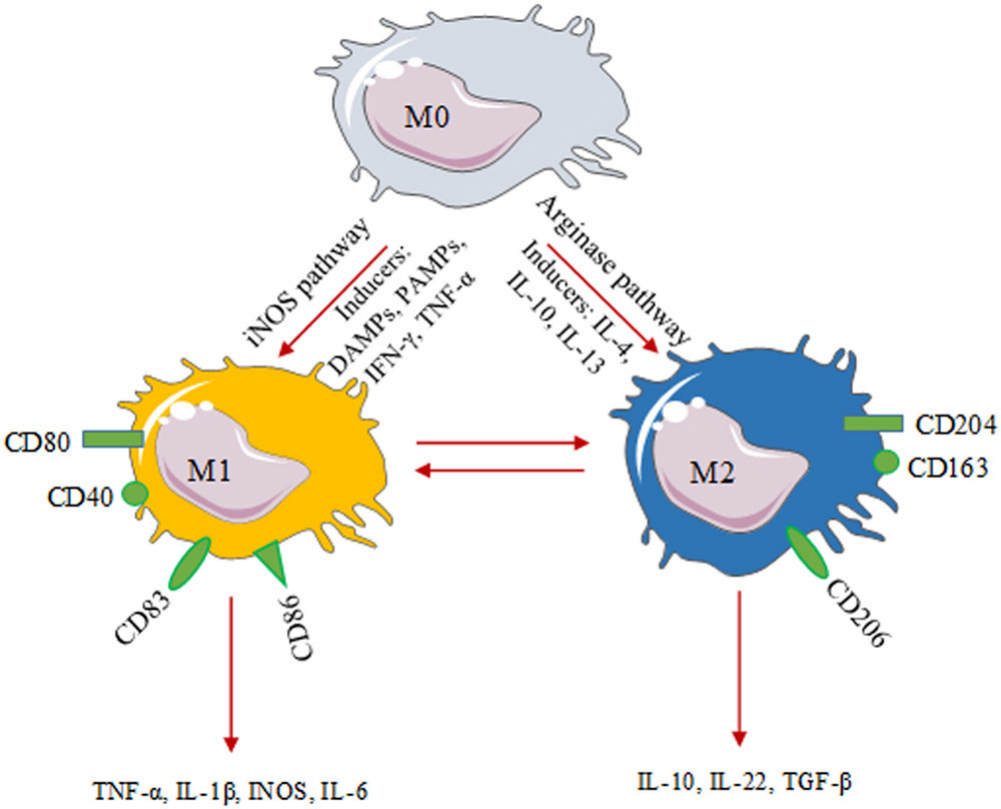
Macrophage polarization and properties

The wide variety of MQ cells' functions occurs as a consequence of their response to diverse microenvironmental stimuli that causes these cells to polarize toward different functional phenotype.^24^ Pathogens infection and tissue injuries lead to MQs polarization toward a “classically activated type 1 (M1)” state, whereas anti‐inflammatory cytokines promote “alternatively activated type 2 (M2),” which regulates inflammation.^25^ M1/M2 functional polarization is dependent on Arginine metabolism through nitric oxide synthase (iNOS) or arginase pathways according to the surrounding signals.^26^ M1 MQs (products of iNOS pathway) activation is accompanied by the potent cytotoxic function and production of pro‐inflammatory cytokines (like TNF‐α, interleukin [IL]‐1β, etc.) and the generation of reactive oxygen and nitrogen products.^27^ M2 MQs, which are the product of the arginase pathway, secrete large amounts of anti‐inflammatory cytokines like IL‐10 and IL‐22 and small amounts of pro‐inflammatory cytokines and express various receptors like the mannose and scavenging receptors, and so on.^28^ Generally, pathogen‐associated molecular patterns (PAMPs), inflammatory cytokines such as tumor necrosis factor (TNF)‐α and interferon (IFN)‐γ, and damage‐associated molecular patterns (DAMPs) induce the M1 phenotype. Conversely, anti‐inflammatory cytokines such as IL‐10, ‐4, and ‐13 induce the M2 phenotype.^29^ CD40, CD80, CD83, and CD86 are used to identify M1 MQs whereas M2 MQs are identified by expressing CD163, CD204, and CD206.^30^ MQs can shift from one phenotype to another in response to different stimulation.^22^ The balance of the M1/M2 phenotype is important in controlling inflammatory conditions.^31^ Throughout the acute inflammatory phase, MQs exhibit the M1 phenotype and induce inflammatory responses. But to suppress the excessive inflammation, MQs polarize to an M2 phenotype with anti‐inflammatory characteristics (Figure 1).^32^

## 3 MACROPHAGE AND GUT INFLAMMATION

Several studies have shown that MQs along with dendritic cells and innate lymphoid cells regulate intestinal homeostasis and barrier integrity. There are numerous resident MQs in the human small and large intestine mucosa.^33^ It is necessary for the healthy gut to distinguish between harmful and harmless antigens and give an appropriate response to them and control inflammation through its powerful mechanisms. Uncontrolled inflammatory responses are known as the cause of some intestinal disorders. It has been suggested that disruptions to MQ abundance and function lead to chronic hyperinflammation accompanied by different GI diseases.^34^ In this regard, the imbalance between pro‐ and anti‐inflammatory cytokines secreted by different types of mucosal immune cells, especially the MQs, is related to the pathogenesis of inflammatory bowel disease (IBD). Increasing the M2 phenotype of MQs is reported as an attractive treatment for IBD.^32^ Postoperative ileus (POI), which is a major clinical problem after abdominal surgery and is known as slowing or stopping GI motility, is reported to be caused by local inflammation initiated by MQs.^34^ Moreover, the GI tract is considered as a major site of human immunodeficiency virus (HIV) infection and mucosal damage in GI tract of HIV‐infected individuals is reported to be caused by intestinal MQs. CD is another inflammatory disorder of the GI tract, in which different immune cells like MQs contribute to its onset or maintenance.^35^

## 4 MACROPHAGE AND CD PATHOGENESIS

Investigations show that gliadin peptides induce the differentiation of the pro‐inflammatory phenotype of MQs (M1 MQ).^36^ In fact, gliadin peptides trigger higher levels of IL‐8 and TNF‐α production by M0 of CD patients. This pro‐inflammatory cytokine secretion is accompanied by a pro‐inflammatory activation state of M0 expressing higher levels of M1 markers, (like CD80, CD86, and CD40) and increased activation of the NF‐κB signaling.^37^ In this regard, Serena et al. in their study observed that gliadin has a pro‐inflammatory effect on both celiac and healthy primary MQs and CD MQs were more responsive to the microenvironment signals than MQs from healthy subjects.^35^ Sedda et al.^38^ also in their study showed the increased expression of the active form of Rapamycin (mTOR), which promotes differentiation of type I MQs, in duodenal biopsy specimens of untreated CD patients.

**Figure 2:**
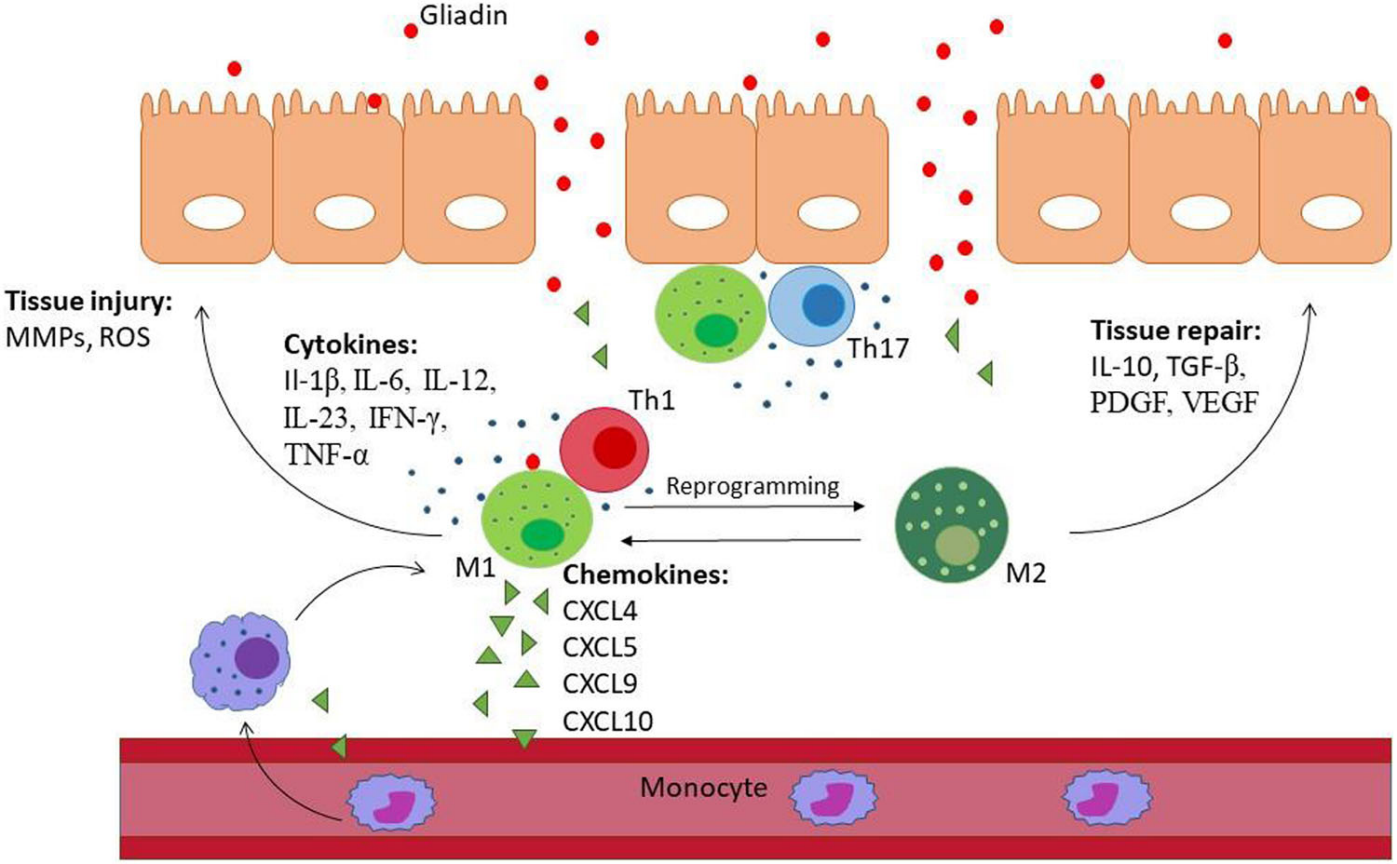
Function of MQ cells in celiac disease pathogenesis. Gliadin peptides stimulate MQ activation toward the pro‐inflammatory phenotype (M1). These cells secrete inflammatory cytokines, which trigger the immune response of Th1 and Th17 cells, as well as recruit other immune cells, including B cells, NK cells, neutrophils, and NKT cells to this site. Moreover, M1 MQs can cause direct tissue damage by producing matrix metalloproteinases (MMPs) and reactive oxygen species (ROS). Consequently, as a result of the activity of M1 MQs and other immune cells and inflammatory responses tissue damages develop. In contrast, anti‐inflammatory MQs (M2 phenotype), which may be produced to reduce inflammation at this site show immunosuppressive and tissue repairing effects by producing cytokines such as IL‐10 and TGF‐β.

Gluten, which is highly resistant to GI digestive proteases, is partially digested into gliadin fragments and passes through the epithelial barrier of the intestinal mucosa and reaches the LP.^39^ During an adaptive immune response, tissue transglutaminase 2 enzyme (tTG2) deamidates gliadin peptides and enhances their binding affinity to HLA‐DQ2/DQ8 molecules expressed on antigen‐presenting cells (APCs). APCs present deamidated immunogenic gliadin peptides to CD4+T cells and activate them, which leads to the adaptive immune response activation and pro‐inflammatory cytokines (like INF‐γ) production.^40^ Dendritic cells (DC) and MQs are the two most known APCs in the intestinal mucosa. CD patients' MQs show high antigen‐presenting ability, which is accompanied by the upregulated expression of the costimulatory molecules CD80, CD86, and CD40, along with higher CD40L expression.^37^ Carlsen et al.^41^ showed that there are several CD68+ tissue MQs in duodenal biopsy samples of CD patients. In the LP, MQs interaction with gliadin peptides leads to myeloid differentiation factor 88 (MyD88)‐dependent pro‐inflammatory cascade that enhances the interaction of T cells with APCs.^42^ MyD88 signaling leads to the activation of NF‐κB in enterocytes, which prompts pro‐inflammatory cytokines transcription contributing to inflammation.^43^ Palova‐Jelinkova et al.^44^ reported that chronic inflammatory enteropathy in CD could be due to the secretion of IL‐1β by monocytes and MQs via the TLR4/MyD88 signaling pathway. In fact, secreted mediators (such as IFN‐γ) from activated T cells may lead to MQs' activation and production of pro‐inflammatory cytokines by them causing mucosal matrix damage (Figure 2).^45^ Moreover, the innate immune system, in which MQs are central to its responses, is involved in the earliest events causing CD pathogenesis.^42^ In more detail, IL‐15 which is the critical mediator of the innate immune response in CD is produced by activated intestinal epithelia and DCs/MQs and induces cytotoxicity of NK cells ultimately leading to intestinal damage in CD.^46^, ^47^

In addition, M2‐related immunosuppressive cytokines were also reported in CD (Figure 2).^48^ For instance, The concentration of IL‐10 is shown to be significantly higher in CD patients' serum samples than in controls.^49^ On this basis, gliadin triggering M2‐like shift of MQs and the expression of arginase 1 and arginase 2 was reported by Barilli et al. study.^50^ On the other hand, protein tyrosine phosphatase non‐receptor type 2 (PTPN2), which is an anti‐inflammatory gene and its deficient was related to the decreased M2 MQs,^51^ was reported to have a loss‐of‐function mutation in CD.^51^

According to the findings, increasing the M2 phenotype of MQs can be an attractive treatment for CD. There are several natural products that can modulate MQ polarization like curcumin, resveratrol, procyanidins, paeonol, and so on. Curcumin is a natural polyphenol derived from turmeric with several pharmacological activities, which has been reported to be able to modulate MQ polarization, mainly through TLR4/MAPK (mitogen‐activated protein kinase)/NF‐κB signaling pathways.^52^ In fact, TLR4 activation is accompanied by inflammatory signaling cascades (like NFkB and MAPK) that result in the production of pro‐inflammatory cytokines (such as IL‐6 and TNF‐α). By inhibiting these pathways curcumin attenuates inflammatory responses and induces M1 to M2 phenotype switching.^53^, ^54^ In an in vitro study, the role of curcumin in inhibiting the polarization of RAW264.7 cells (M0) to M1 phenotypes and switching M1 or M0 MQs to M2 phenotype, has been demonstrated.^55^ Resveratrol (3,5,4′‐trihydroxystilbene) is a natural polyphenolic phytoalexin derived from a wide range of plants including peanuts, grapes, and berries.^56^ Resveratrol has been shown to have potent anti‐inflammatory and antioxidant activity.^57^ In a recent in vitro study, it has been reported that resveratrol could induce MQs to differentiate into M2 phenotype by affecting the TLR4/MyD88 receptor pathway.^58^ It can also decrease the expression of M1 related pro‐inflammatory cytokines like TNF‐α, IL‐1β, and IL‐6.^59^ Procyanidins are polyphenols with anti‐inflammatory activity, which are widely found in cinnamon, apples, grapes, and cocoa beans.^60^ According to a recent report, procyanidin B2 (PCB2) induces M2 MQs polarization through peroxisome proliferator‐activated receptor γ (PPARγ) signaling activation.^61^ Paeonol (2′‐hydroxy‐4′‐methoxyaeetophenone) is a simple phenolic compound of peonies like *Paeonia suffruticosa* with multiple anti‐inflammatory effects. Inhibitory effects of paeonol on M1 MQs polarization have been reported in recent studies.^62^, ^63^

There are also some medical compounds with MQs modulatory activity. Dexamethasone is a glucocorticoid that is widely used as an anti‐inflammatory drug and can promote MQs polarization toward the M2 phenotype.^64^ Jeong et al. demonstrated that THP1 monocytes treatment with dexamethasone induced M2 polarization and IL‐10 and TGF‐β production.^65^ Moreover, it has been shown that Niacin, known as vitamin B3, which is generally used as a nutritional supplement, by increasing prostaglandin (PG) D2 release in MQs and activating its receptor (PD1) results in MQs polarization shift toward the M2 phenotype.^66^ Azithromycin, which is an antibiotic medication that fights a wide variety of bacteria, is reported to be able to polarize MQs to M2 phenotype and inhibit M1 related pro‐inflammatory cytokines (like IL‐12 and IL‐6) and proteins (like CCR7 and IL‐12p70) and increase M2 related proteins (like IL‐10 and CCL18) expression.^67^ Furthermore, according to the reports, by suppressing AP1/NFκB and MAPK signaling pathways and activating PI3K/Akt and STAT3 signaling pathways, strain‐specific probiotics can also increase M2 MQs.^68^ Tortora et al.^69^ in their recent study demonstrated that the administration of JWH‐133 [100 nM] as an agonist of cannabinoid receptor 2 (CB2), a receptor with important anti‐inflammatory and immunoregulatory properties that affects immune cells activation, induced CD patients' peripheral blood isolated MQs polarization toward the anti‐inflammatory M2 phenotype.^69^ These reports point to the importance of paying attention to finding an M1/M2 modulator with the least complications to be used as a complementary therapy for CD patients.

## 5 CONCLUSION

According to the results of published studies that we have discussed, both M1 and M2 MQs are present in the small intestine of CD patients. However, CD patients present an imbalanced ratio and an impaired transition from pro‐inflammatory to anti‐inflammatory responses due to inappropriate responses to gliadin peptides. The M1/M2 MQs polarization balancing regulators can be considered novel therapeutic targets for CD.

## AUTHOR CONTRIBUTIONS

*Mohammad Rostami‐Nejad, Kaveh Baghaei, Abbas Yadegar*: Designed the study. *Sara Molaaghaee‐Rouzbahani, Nastaran Asri*: Conducted the research. *Sara Molaaghaee‐Rouzbahani, Nastaran Asri, Somayeh Jahani‐Sherafat*: Wrote the paper. *Davar Amani, Mohammad Rostami‐Nejad, Andrea Masotti, Hamed Mirjalali*: Revised the paper. All authors read and approved the final manuscript.

## Data Availability

Data sharing is not applicable to this article as no new data were created or analyzed in this study.
